# Administration of anti-HER2 antibody after nonmyeloablative allogeneic stem cell transplantation in metastatic breast cancer

**DOI:** 10.1038/sj.bjc.6603114

**Published:** 2006-04-25

**Authors:** G L Banna, S M L Aversa, G Crivellari, C Ghiotto, V Chiarion-Sileni, S Monfardini

**Affiliations:** 1 Division of Medical Oncology, Istituto Oncologico Veneto, Via Gattamelata 64, Padova 35100, Italy

**Sir,**

We read with great interest the article by Arnould *et al* (2006). In their study, they investigated by immunohistochemistry (IHC) immune cell response during neoadjuvant primary systemic therapy with trastuzumab and docetaxel in patients with IHC 3+ HER2-positive primary breast cancer. Trastuzumab treatment was associated with significantly increased numbers of tumour-associated NK cells and lymphocyte expression of granzyme B and TiAI compared with controls. This suggests that trastuzumab plus taxanes lead to enhanced NK cell activity, thus confirming that NK cell activity via antibody-dependent cellular cytotoxicity (ADCC) is one of the mechanisms of action of trastuzumab, as similarly reported *in vivo* by other authors (Repka *et al*, 2003; Gennari *et al*, 2004). Furthermore, this observation may partially account for the synergistic activity of trastuzumab and docetaxel in breast cancer. In fact, taxanes lead to increased serum concentrations of some cytokines and enhancement of NK cell activity (Tsavaris *et al*, 2002).

Moving from the same considerations, along with the observation that graft-versus-tumour responses in metastatic breast cancer amount to 25–40% following nonmyeloablative allogeneic haemopoietic stem cell transplantation (NST) (Bishop, 2004), and that recognition by donor immune cells of minor histocompatibility antigens and tumour-specific and/or overexpressed antigens is the theoretical background for these alloimmune responses, we treated two patients with IHC 3+ HER2-positive metastatic progressive breast cancer with weekly trastuzumab administrations (4 mg kg^−1^ intravenously (i.v.) the first dose, 2 mg kg^−1^ i.v. the other doses) in case of disease progression (PD) following NST. In fact, we hypothesised that the use of antitumoral monoclonal antibodies (moAbs) (such as trastuzumab) following NST might enhance the allogeneic ADCC against tumour cell targets, possibly avoiding undesirable graft-versus-host reactions; conversely, a cellular component might enhance the effects of antitumoral moAbs and vaccines directed against the HER2 antigen (Reilly *et al*, 2001; Disis *et al*, 2002; zum Buschenfelde *et al*, 2002; Wolpoe *et al*, 2003). Patients were treated in a clinical trial approved by an independent ethical committee and in accordance with the Helsinki Declaration. Written informed consent was obtained from the two patients before enrolment.

Patients were aged 40 and 49 years, respectively. Both were heavily pretreated with five and three chemotherapy lines (including docetaxel), respectively. Trastuzumab, radiotherapy and several hormonal treatments were administered to both patients and both had PD at the time of transplant; the first one had bone and liver metastases, and the second liver metastases.

Doses of 8.0 and 5.0 × 10^6^ kg^−1^ lenograstim-stimulated CD34+ cells were given to the two patients, respectively, in both cases from an HLA-identical sibling (on day 0) following a reduced-intensity conditioning with thiotepa 10 and 5 mg kg^−1^ i.v., cyclophosphamide 120 mg kg^−1^ i.v. and fludarabine 120 mg m^−2^ i.v. Graft-versus-host disease (GVHD) prophylaxis consisted of cyclosporin A and short-course methotrexate.

Neutrophils and platelets engraftment were rapidly achieved on days +13/+10 and +9/+11 after NST, respectively. Stable full donor chimerism (FDC) (by PCR analysis) on total cells was reached on day +28 in both patients. On separated peripheral myeloid and lymphoid cells, stable FDC was achieved on day +28 in the first patient and on days +130 and +170, respectively, in the second patient. Lymphoid FDC was reached following the first donor lymphocyte infusion (DLI) on day +140 in the second patient.

The first patient started weekly trastuzumab administrations on day +48, because of early liver PD. However, no disease response and/or GVHD were observed and the patient eventually died of liver PD 6 months later.

The second patient developed on day +71 grade 3 liver failure (according to NCI criteria v3.0) with jaundice, in the absence of other clinical signs of acute GVHD but with liver and right breast PD evidence. The patient was hospitalised and weekly trastuzumab was started. Trastuzumab resulted in a rapid decrease of serum bilirubin, transaminases, jaundice and hepatomegaly. Right breast recurrence disappeared, but subsequent liver PD was shown by a CT scan on day +98. Two DLIs were then administered on day +140 (dose of 1 × 10^6^ CD3+ kg^−1^) and on day +178 (dose of 1 × 10^7^ CD3+ kg^−1^), and 24 h later, the 10th and the 14th trastuzumab administration, respectively. On day +154, left sovraclavear nodes and right breast PD, along with an increase of CEA and CA15.3 serum markers and the development of a grade 4 liver failure occurred. A Kehr drain was positioned. On day +195, weekly paclitaxel 60 mg i.v. was added to trastuzumab, resulting in liver partial remission (PR) on day +288 (Figure 1) and clinical remission of left sovraclavear, right breast relapse and normalization of serum tumour markers. Kehr's drain was removed on day +364. No signs of chronic GVHD were observed. The patient remained well and in PR until day +405, when liver PD reoccurred. Chemotherapies with gemcitabine, carboplatin and capecitabine were sequentially added to trastuzumab, but they were ineffective and the patient died on day +658. Of note, an increase of CD8+CD3+ cytotoxic T cells and CD16+CD56+ NKs was observed after NST by immunophenotypical blood T- and B-cell analysis, and a CD19+ cells peak was registered after the start of trastuzumab (see Table 1).

Even though this is not direct evidence to support the role of an allogeneic ADCC, the regression of severe liver impairment owing to liver PD by the administration of trastuzumab after NST, the subsequent disease response by the addition of paclitaxel to trastuzumab in a patient who had been heavily pretreated with several drugs including docetaxel and trastuzumab, and the concomitant increase of NK cells and CTLs in peripheral blood might support the observation of Arnould *et al* (2006) and suggest further researches to validate the hypothesis of a synergistic cytotoxicity with combined immuno- and chemotherapy.

## Figures and Tables

**Figure 1:**
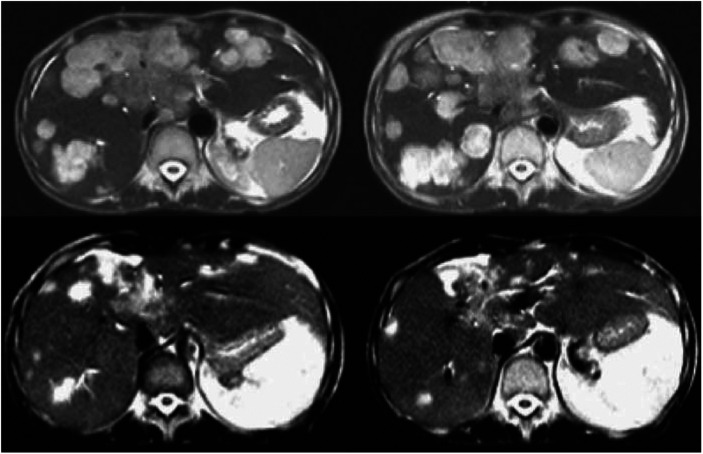
Abdomen T2-weighted MRI of day +220 after NST showing liver disease progression (upper images). Partial disease remission on day +288 after the addition of paclitaxel to trastuzumab (lower images).

**Table 1:** Blood T- and B-cell immunophenotypical analysis

Day after NST		+30	+60	+90	+135	+165	+250
WBC (*μ*l)		2750	13 800	7150	4880	5710	2670
Lymphocytes (*μ*l)		750	170	350	680	2450	860
CD3+ (T cells) (%)	(68–82)	50	44	60	57	90	75
CD4+CD3+ (Th/ind) (%)	(36–52)	34	24	41	14	8	20
CD8+CD3+ (Ts/CTL) (%)	(20–34)	12	10	14	38	81	48
CD19+ (B cells) (%)	(5–16)	1	1	12	2	1	4
CD16+CD56+ (NK) (%)	(1.5–15)	43	31.3	25.7	39.7	9.5	20.9
CD3+CD16+							
CD56+ (Non-MHC-restr. CTLs) (%)	(1–9.7)	6	3	4	2	2	2
CD4+/CD8+	(1.5–2.1)	2.8	2.4	2.9	0.4	0.1	0.4

CTL, cytotoxic T-lymphocyte; MHC, major histocompatibility complex; NK, natural killer cells; Non-MHC-restr, non-major histocompatibility complex-restricted; NST, nonmyeloablative allogeneic haemopoietic stem cell transplantation; WBC, white blood cell.
